# The Comparison between Endoscopic Submucosal Dissection and Surgery in Gastric Cancer: A Systematic Review and Meta-Analysis

**DOI:** 10.1155/2018/4378945

**Published:** 2018-02-18

**Authors:** Junbi Hu, Yan Zhao, Mudan Ren, Yarui Li, Xinlan Lu, Guifang Lu, Dan Zhang, Dake Chu, Shuixiang He

**Affiliations:** Department of Gastroenterology, The First Affiliated Hospital of Xi'an Jiaotong University, Xi'an, Shaanxi 710061, China

## Abstract

**Aims:**

There are two treatment modalities for early gastric cancer (EGC)—surgery and endoscopic submucosal dissection (ESD). We aimed to compare the safety and efficacy of ESD with surgery.

**Method:**

The article was performed by searching PubMed databases. Data were extracted using predefined form and odds ratios (OR) with 95% confidence intervals (CI) calculated and *P* value.

**Results:**

13 studies were identified. The incidence of perforation in two groups was different [OR = 6.18 (95% CI: 1.37–27.98), *P* = 0.02]. The prevalences of synchronous and metachronous cancer in the ESD group were higher than those in the surgery group [OR = 8.52 (95% CI: 1.99–36.56), *P* = 0.004 and OR = 7.15 (95% CI: 2.95–17.32), *P* < 0.0001]. The recurrence and complete resection rates were different [OR = 6.93 (95% CI: 2.83–16.96), *P* < 0.0001 and OR = 0.32 (95% CI: 0.20–0.52), *P* < 0.00001]. Compared with the surgery group, the hospital stay was shorter [IV = −7.15 (95% CI: −9.08–5.22), *P* < 0.00001], the adverse event rate was lower, and the quality of life (QOL) was better in the ESD group. The difference of bleeding was not found.

**Conclusion:**

ESD appears to be preferable for EGC, due to a lower rate of adverse events, shorter hospital stay, cheaper cost, and higher QOL.

## 1. Introduction

Gastric cancer is the fourth most common malignancy and the second leading cause of cancer mortality worldwide [1]. The prognosis of gastric cancer is significantly depended on the early diagnosis. With the development and widespread implementation of endoscopic techniques, such as chromoendoscopy, narrowband imaging, magnifying endoscopy, and confocal microscopy, the diagnosis rates of patients with early gastric cancer (EGC) have been increasing [2]. The EGC refers to the lesion confined to the mucosa and submucosa, regardless of lymph node metastasis (LNM) [3].

There are several treatment options for EGC, such as the endoscopic mucosal resection (EMR), endoscopic submucosal dissection (ESD), and gastrectomy plus D1 or D2 lymphadenectomy through laparoscopic or open operation. Among these treatment methods, radical surgery can achieve adequate oncological clearance with wide resection margins, nodal dissection, and low rate of recurrence. However, the perioperative mortality, compromised long-term gastrointestinal function, long operation time, and lower quality of life after surgical resection could not be ignored [4]. In recent years, with the development of endoscopic technology, the ESD gradually becomes the main choice for EGC. However, the technology of ESD is so difficult that the complications were accompanied [5]. In addition, the impossibility of regional lymph nodes removed during the ESD procedure is another major limitation, which may probably result in tumor recurrence and the invasive radical gastrectomy. Previous systematic review and meta-analysis revealed that the 5-year DFS survival rate may not be different between the endoscopic resection and surgery in the treatment of EGC as well [6].

Based on these investigations, we could find that both ESD and surgery have advantages and disadvantages. It is still uncertain which therapy could be better for EGC. Therefore, we performed this systematic review and meta-analysis to compare the safety and efficacy of ESD with surgery for early gastric cancer.

## 2. Methods

The present systematic review and meta-analysis was performed according to a protocol determined before the study, including search strategy, selection of article, and statistical analysis.

### 2.1. Search Strategy

A PubMed search was carried out using terms ((((ESD OR endoscopic submucosal dissection))) AND (((gastric cancer OR gastric carcinoma OR stomach cancer OR intraepithelial neoplasia)))) AND ((operation OR surgery OR surgical OR resection)). The search was performed on November 4, 2016. When the same data was reported in more than one published paper, only the studies, with more complete data and a more extensive interval of enrolment, were included in our review and meta-analysis.

### 2.2. Selection of Article

Article selection was determined by four inclusion criteria:

- Only adults were included in the study.
- Only patients diagnosed with EGC were included regardless of lymph node metastasis.
- The studies definitely included two groups, the endoscopic submucosal dissection and surgery groups, and there were no limitations in gastrectomy or laparoscopic resection.
- Articles published in English were included.

We excluded the following: editorials; comments; letters to the editor; review articles; case reports; guidelines articles; animal studies; number of patents in any included studies less than ten; studies including patients with other malignancies, such as esophageal carcinoma, colorectal cancer, and polyps; articles without explicit data on the ESD group, but rather endoscopic resection (ER); and the articles with insufficient data.

### 2.3. Data Extraction, Outcome Measures, and Quality Assessment

All data were extracted independently by two reviewers (Hu J. and Zhao Y.). In case of disagreement between the two reviewers, a consensus was achieved through discussion among the two reviewers. The primary outcome was complication which refers to perforation and bleeding in our study. The secondary outcome was synchronous cancer, metachronous cancer, complete resection, adverse events, hospital stay, hospital cost, quality of life, 5-year disease-free survival (DFS) rate, and 3-year survival rate. The quality of included studies was assessed by the Newcastle–Ottawa Scale (NOS) [7, 8].

### 2.4. Definitions

The surgery group included traditional gastrectomy and laparoscopic gastrectomy. Complications in our study refer to bleeding and perforation. Bleeding can be subdivided into immediate (intraoperative) bleeding and delayed bleeding. The significant immediate bleeding is defined as the hemoglobin (Hb) reduced more than 2 g/dL comparing preprocedure and next-day levels [5].

Metachronous cancer refers to a newly developed cancer after at least 1 year after ESD or surgery. Synchronous cancer refers to the malignant lesions distinctly separated by a microscopically normal gastric wall and ruled out from local extension or metastasis [9]. Complete resection refers to resection tumors complete with negative tumor margins. The length of hospital stay refers to the time from the date of ESD or surgery to the discharge date.

### 2.5. Statistical Analysis

Data was analyzed using Review Manager (version 5.0). Mean, standardized deviations (SD), and 95% confidence interval (CI) were calculated for continuous data; odds ratio (OR) or risk ratio (RR) with 95% CI was calculated for dichotomous data. Heterogeneity was assessed by using chi-squared test (*P* < 0.10 was considered to represent significant statistical heterogeneity) and *I^2^* statistic (*I^2^* higher than 50% was considered as having substantial heterogeneity) [10]. The random effects models were chosen for study with *I^2^* higher than 50%, and the fixed effects models were chosen for study groups with *I^2^* lower than 50%. The combined odds ratios (OR) were calculated by the Mantel-Haenszel model. The publication bias was evaluated by using the Funnel plots. For all analyses, *P* < 0.05 was considered statistically significant.

## 3. Results

### 3.1. Description and Quality Assessment of Included Studies

**Figure 1:**
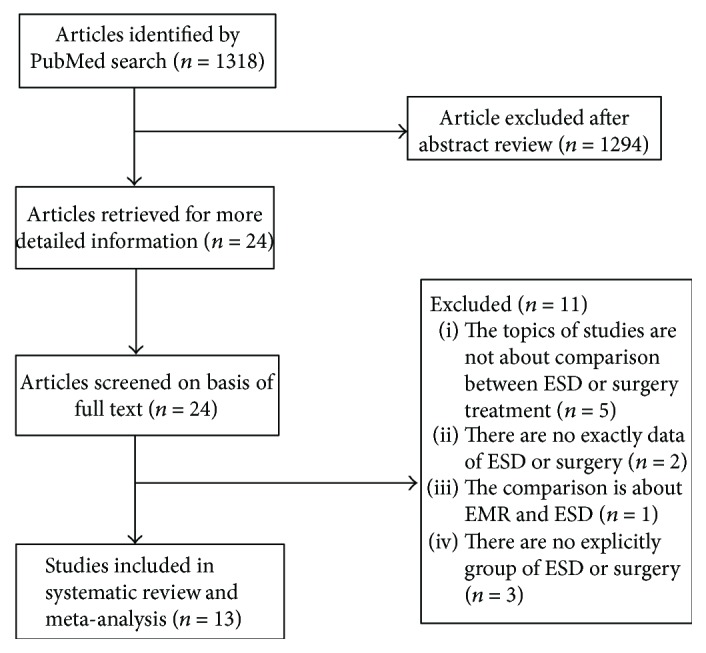
Flow chart used in study. ESD: endoscopic submucosal dissection.

The search strategy initially identified a total of 13 studies (Figure 1). The characteristics of these studies and a corresponding characteristic of included 2106 patients are summarized in Table 1 [4, 11–22]. Among these studies, 12 studies were, respectively, case-control study, and another study was cross-sectional study [4, 11–22]. Four studies applied the propensity score-matched analysis to avoid analytical bias. In most studies, the endpoints included 5-year DFS or 3-year survival rate, complications, recurrence rate, synchronous cancer, metachronous cancer, and medical adverse events. In one study, the main objective was to compare the quality of life (QOL) in EGC patients who underwent endoscopic submucosal dissection (ESD) or surgery [13]. There was a study devoting to analyze the differences of medical costs between ESD and surgery [16].

### 3.2. Comparison of Complications

#### 3.2.1. Bleeding

**Figure 2:**
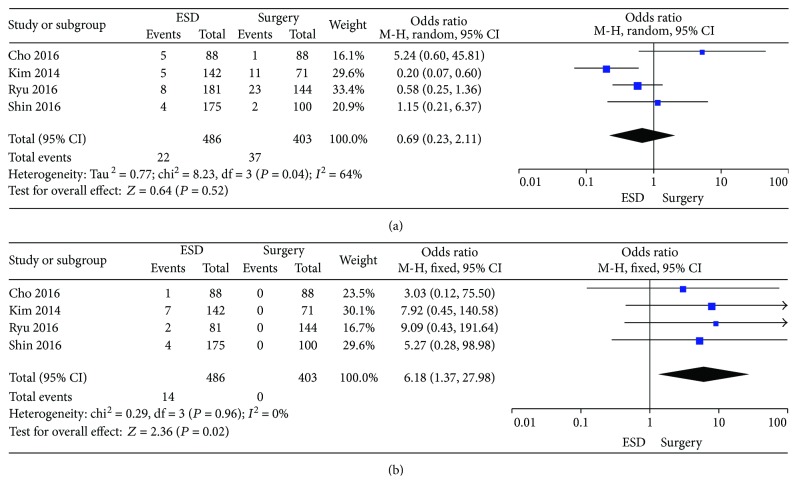
Forest plots showing the results of meta-analysis comparing the prevalence of bleeding and perforation between patients with ESD and surgery. (a) Comparing the prevalence of bleeding between ESD and surgery. (b) Comparing the prevalence of perforation between ESD and surgery. ESD: endoscopic submucosal dissection.

The bleeding data in these studies included procedure-related and postprocedure bleeding, which were identified in 4 studies [12, 15, 19, 20]. The high heterogeneity can be initially found in these studies (*P* = 0.04; *I*^2^ = 64%) (Figure 2(a)). The random effects model was applied. There was no statistical difference between the ESD group and the surgery group [OR = 0.69 (95% CI: 0.23–2.11), *P* = 0.52]. Then, sensitivity analysis was performed by using a funnel plot. After excluding the study which was obviously different from others, the heterogeneity becomes lower (*P* = 0.17; *I*^2^ = 44%). In the study conducted by Kim et al., we summed the data of guideline criteria bleeding and expanded criteria bleeding together [15].

#### 3.2.2. Perforation

Data of perforation were reported in 4 studies [12, 15, 19, 20]. There was no heterogeneity in these studies (*P* = 0.96; *I*^2^ = 0%). The fixed effects model was applied. The perforation rate in the ESD group (2.9%) is higher than that in the surgery group (0%) [OR = 6.18 (95% CI: 1.37–27.98), *P* = 0.02] (Figure 2(b)).

### 3.3. Comparison of Recurrence Rate

**Figure 3:**
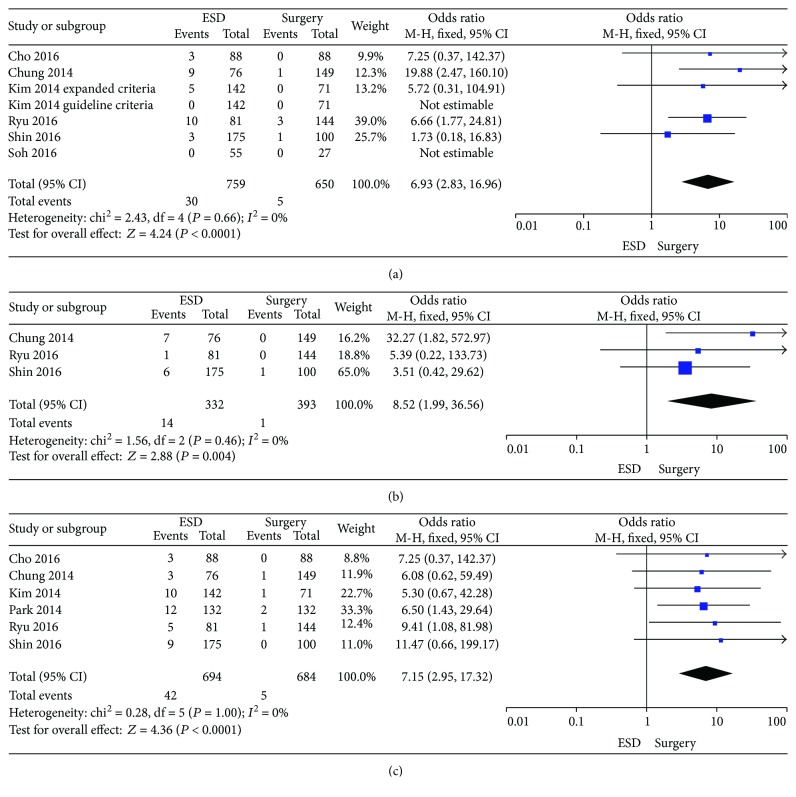
Forest plots showing the results of meta-analysis comparing the prevalence of recurrence, synchronous cancer, and metachronous cancer between patients with ESD and surgery. (a) Comparing the prevalence of recurrence between ESD and surgery. (b) Comparing the prevalence of synchronous cancer between ESD and surgery. (c) Comparing the prevalence of metachronous cancer between ESD and surgery. ESD: endoscopic submucosal dissection.

There were six studies which reported the data of recurrence in the ESD and surgery groups [12, 14, 15, 19–21]. The recurrence rate in the ESD group (4.0%) is higher than that in the surgery group (0.8%). The OR was 6.93 (95% CI: 2.83–16.96, *P* < 0.0001), without a significant heterogeneity (*I*^2^ = 0%, *P* = 0.66). The fixed effects model was applied (Figure 3(a)).

### 3.4. Comparison of Synchronous Cancer

Three studies reported the data of synchronous cancer [14, 19, 20]. The prevalence of synchronous cancer in the surgery group (0.3%) was significantly lower than that in the ESD group (4.2%). The OR for synchronous cancer was 8.52 (95% CI: 1.99–36.56, *P* = 0.004), without a significant heterogeneity (*I*^2^ = 0%, *P* = 0.46). Thus, fixed effects model was applied (Figure 3(b)).

### 3.5. Comparison of Metachronous Cancer

The data could be extracted from six studies [12, 14, 15, 18–20]. There was no heterogeneity showing (*P* = 1.00; *I*^2^ = 0%), and then, the fixed effects model was applied. The prevalence of metachronous cancer in the ESD group (6.1%) was significantly higher than that in the surgery group (0.7%). The OR for metachronous cancer was 7.15 (95% CI: 2.95–17.32, *P* < 0.0001) (Figure 3(c)).

### 3.6. Comparison of Complete Resection

**Figure 4:**
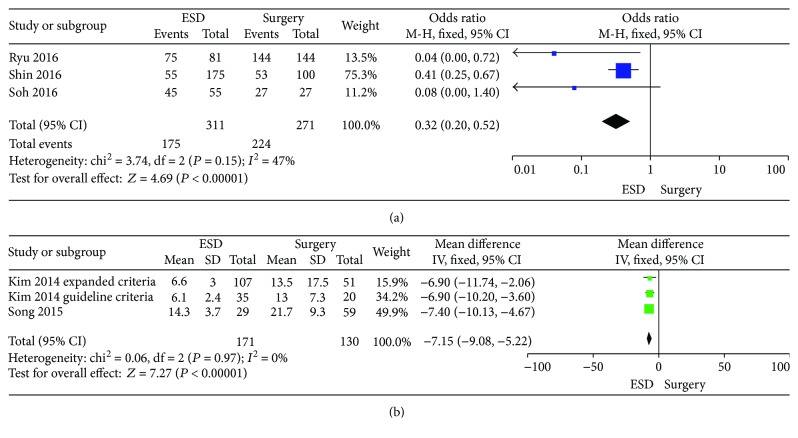
Forest plots showing the results of meta-analysis comparing the complete resection rate and hospital stay between patients with ESD and surgery. (a) Comparing the complete resection rate between ESD and surgery. (b) Comparing hospital-stay between ESD and surgery. ESD: endoscopic submucosal dissection.

The data of complete resection among 3 studies were identified [19–21]. The rate of complete resection in the ESD group (56.3%) was lower than that in the surgery group (82.7%). The OR for complete resection was 0.32 (95% CI: 0.20–0.52, *P* < 0.001), without a significant heterogeneity (*I*^2^ = 47%, *P* = 0.15). Thus, the fixed effects model was applied (Figure 4(a)).

### 3.7. Comparison of Hospital Stay

In 7 studies, the hospital stay time was reported [4, 11, 15, 18–21]. There were two studies using the approach “mean ± SD” describing the data, which were brought into meta-analysis [4, 15] (Figure 4(b)). The IV was −7.15 (95% CI: −9.08 to −5.22, *P* < 0.001), without a significant heterogeneity (*I*^2^ = 0%, *P* = 0.97). The fixed effects model was applied. The other 4 studies used the approach “mean range” describing the data, and one study used the “mean” as the approach. All these studies suggested that there were significant statistic differences of hospital stay time between the ESD and the surgery groups. Compared with the surgery group, the hospital stay time in the ESD group was much shorter.

### 3.8. Comparison of Survival Rate

There were eight studies describing the survival rate of patients treated with ESD and surgery, and the results are shown in Table 2 [11, 12, 15, 17–20, 22]. Among these eight studies, we could find that the 5-year DFS rate in the surgery group was significantly higher than that in the ESD group [19]. One study showed that the 3-year survival rate was significantly higher in the surgery group than in ESD group [18]. However, the meta-analysis for 5-year DFS rate or 3-year survival rate could not be performed due to the data unavailable in the enrolled studies.

### 3.9. Comparison of Hospital Costs

The hospital cost in ESD and surgery showed significant differences among 3 studies. In general, ESD has lower medical costs than conventional surgeries for EGC when it is done in conservative indication [16]. In a study conducted by Shin et al., the total cost of hospitalization (between 2012 and 2013) for ESD, subtotal gastrectomy, and total gastrectomy were approximately $1871, $5925, and $6476, respectively [20]. Another study conducted by Soh et al. suggested that the significant statistic difference existed in the cost of ESD [$2374 (interquartile range (IQR) 1858–3016)] and surgery [$4954 (IQR 4285–5918)] (*P* ≤ 0.0001), respectively.

### 3.10. Comparison of Adverse Events

Among 13 studies, two studies reported the status of adverse events with ESD and gastric cancer treatment [18, 22]. In one study, adverse event rates of the ESD group were significantly lower compared with those of the surgery group. In both groups, there were no procedure-related mortalities observed. Two cases were with in-hospital mortality because of anastomotic leakage in the surgery group [22]. The other study showed that the incidence of total medical and surgical adverse events was not significantly different. But there were 2 patients (1.5%) who died after surgery (1 with splenic artery bleeding and 1 with anastomosis site leakage) [18].

### 3.11. Comparison of Quality of Life

There are two articles comparing the differences of the quality of life between the ESD and the surgery groups [4, 13]. They both used the Short-Form Health Survey and the European Organization for Research and Treatment of Cancer QOL questionnaires (QLQ-C30 and EORTC-QLQ-STO22) [23, 24]. They confirmed that the ESD for EGC provided a better quality of life than surgery, and they found that fatigue, nausea/vomiting, loss of appetite, constipation, reflux, body image, eating restrictions, and so forth reached significant statistical difference between these two groups. These symptoms were more common in the surgery group.

## 4. Discussion

The purpose of this study is to compare the efficacy between the ESD and surgery. After retrospectively analyzing enrolled researches, we could find that ESD would be preferable to surgery in terms of lower occurrence rate of adverse events, shorter hospital stay, cheaper cost, and higher QOL for the treatment of EGC, while surgery is advanced in higher complete resection rate, higher 5-year DFS rate, and 3-year survival rate.

According to our analysis, compared to those in the ESD group, the 5-year DFS rate and 3-year survival rate in the surgery group are higher, which may be affected by the interval of endoscopic surveillance and the follow-up periods. The interval of endoscopic surveillance was not definitely established, and the median follow-up periods in the ESD group and surgery group were relatively short. What is worse, the follow-up period was shorter in the ESD group than in the surgery group [18]. Thus, the short-term follow-up endoscopic surveillance and the longer follow-up periods may be needed to verify this conclusion. More clinical research and meta-analysis are still needed as well.

Bleeding, one of the most serious complications of ESD, is subdivided into immediate (intraoperative) bleeding and delayed bleeding. In our study, there was no statistically significant difference between the ESD group and surgery group. It was reported that the rate of delayed bleeding after ESD ranged from 0% to 15.6% [5]. Due to the development of technology, ESD-related bleeding could be treated well. The electrocautery, cutting device, and water flushing are modalities solving problems when the vessels or the exact bleeding point is found during procedure [5, 25]. Endoscopic clips or electrocautery using hemostatic forceps can be applied in the early days of delayed bleeding, and injection method is preferable in the later days of delayed bleeding. Perforation is another critical and common complication, which is related to lesion size, location, ulcer finding, deep invasion, and technical expertise. The risk of perforation reportedly ranges from 1.2% to 5.2% for ESD treatment [5]. Once happening, there are several methods to deal with the perforation effectively, such as endoscopic closure with endoscopic clips, peritoneal tap, and usage of CO_2_ insufflation [26, 27].

Tumor recurrence is an unignorable problem in the treatment for EGC, which can recur as synchronous cancer or metachronous cancer. There was a study reporting that the incidence of metachronous or synchronous tumor was 4.8% and 1.2% per person-year in the differentiated and undifferentiated cancer groups [28]. In our study, the incidence of metachronous cancer was higher in patients who underwent ESD (6.1%) than in those who underwent surgery (0.73%), and the incidence of synchronous cancer was higher in patients who underwent ESD (4.2%) than in those who underwent surgery (0.25%) as well. In order to solve the problems, annual or biannual surveillance esophagogastroduodenoscopy (EGD) and abdominal computed tomography (CT) might be necessary for EGC with absolute and expanded indications at least 5 years after curative ESD to detecting the progress of gastric cancer. Even the second endoscopic resection and additional gastrectomy may be applied to solve this problem.

It was known that the complete resection is one of the advantages of ESD compared to endoscopic mucosal resection (EMR) [29]. In our analysis, we find that the rate of complete resection in the ESD group (56.3%) was lower than that in the surgery group (82.7%). The risk factors of incomplete resection are the tumor size > 20 mm, undifferentiated-type, submucosa invasion, and lesion location of upper/middle in positive lateral margins (LM) or positive vertical margins (VM). The incidence of incomplete resection may be related to misdiagnosis. If endoscopic technique is improved and awareness of endoscopists is enhanced, the rate of misdiagnosis is decreased and the incomplete resection would be decreased as well [30]. In addition, surgery may be an alternative or second-line treatment when ESD is not curative.

This systematic review and meta-analysis had several limitations. Firstly, there was no randomized controlled trial published. Most of the studies failed to provide detailed information about the survival outcomes. Thus, the meta-analysis about the survival results, hospital cost, and the quality of life between these two groups were not performed. Secondly, in the current systemic review, 12 articles were conducted in Asian countries, and most of them were from Korea. On this side, the data of our study was lacking of representative in the worldwide range.

In conclusion, the present systematic review and meta-analysis suggested that, compared with surgery, ESD would be preferable for patients with EGC, due to lower rate of adverse events, shorter hospital stay, lower cost, and higher QOL. Further randomized controlled and multicentral clinical studies with large samples from additional countries are needed to confirm these findings and compare the efficacy of ESD with surgery.

## Figures and Tables

**Table 1:** Summary of published studies and the characteristic of patients.

Author	Country	Year	Study design	Article type	No. of patients	Median age (years)	Gender (male)	Depth of invasion (%) SM1	Tumor size (mm)	Follow-up (months)	Quality score
Chiu	China	2012	Retrospective	Full text	ESD 74/surgery 40	ESD66.3 (14–88)/surgery 67.0 (33–84)	ESD 66.2%/surgery 57.5	ESD 10.8%/surgery 52.5%	ESD 18.5 (8–40)/surgery 24.7 (10–40)	ESD 27.0 (range 1–65)/surgery 77.6 (range 1–180)	7
Cho	Korea	2016	Retrospective	Full text	ESD 88/surgery 88	ESD (61.8 ± 9.8)/surgery (61.3 ± 9.8)	ESD 71.6%/surgery 70.5	ESD 17.0%/surgery 20.5%	ESD 21.8 ± 12.1/surgery 21.4 ± 10.1	ESD 77 (range 18–107)/surgery 78 (range 1–113)	7
Choi	Korea	2015	Cross-sectional	Full text	ESD 137 /surgery 188	ESD 67.2 (SD 9.9)/surgery 61.4 (SD 11.5)	ESD 75.2%/surgery 67.0%	ESD 4.4%/surgery 15.4%	N/A	N/A	N/A
Chung	Korea	2014	Retrospective	Full text	ESD 76/surgery 149	ESD (60.1 ± 13.2)/surgery (56.7 ± 12.8)	ESD 75.2%/surgery 67.0%	N/A	ESD ≤ 10 (18.4%), >10 ≤ 20 (57.9%), >20 ≤ 40 (19.7%), >4 (3.9%)/surgery ≤ 10 (27.5%), >10 ≤ 20 (72.5%), >2 ≤ 40 (0%), >4 (0%)	ESD 41.7 ± 22.6 (SD)/surgery 42.8 ± 17.3 (SD)	7
Kim	Korea	2014	Retrospective	Full text	ESD 142/surgery 71	ESD (62.0 ± 10.3)/surgery (56.7 ± 12.0)	ESD 66.2%/surgery 81.7%	ESD 4.9%/surgery 12.7%	ESD ≤ 10 (16.9%), ≤20 (72.7%), ≤30 (21.1%), >30 (12.7%)/surgery ≤ 10 (15.5%), ≤20 (54.9%), ≤30 (14.1%), >30 (15.5%)	ESD 76.7 ± 16.5 (SD)/surgery 65.5 ± 16.5 (SD)	7
Najmeh	Canada	2016	Retrospective	Full text	ESD 30/surgery 37	ESD74 (40–86)/surgery 75 (34–86)	ESD 77%/surgery 65%	N/A	ESD 1.8 (0–5)/surgery 2.8 (0–9)	ESD 74 (range 40–86)/surgery 75 (range 34–86)	7
Park	Korea	2014	Retrospective	Full text	ESD 132/surgery 132	ESD (73.9 ± 3.5)/surgery (74.4 ± 3.7)	ESD 73.5%/surgery 66.7%	ESD 6.8%/surgery 6.8%	ESD < 10 (39.4%), 10–20 (40.9%), 20–30 (13.6%), >30 (6.1%)/surgery < 10 (34.1%), 10–20 (43.2%), 20–30 (13.6%), >30 (9.1%)	ESD 17.6 (range 9.9–25.7)/surgery 24.2 (range 12.5–36.7)	7
Ryu	Korea	2016	Retrospective	Full text	ESD 81/surgery 144	ESD (63.65 ± 8.57)/surgery (61.37 ± 9.50)	ESD 72.8%/surgery 81.9%	N/A	ESD 19.32 ± 11.31/surgery 20.55 ± 10.68	ESD 78.12 ± 9.72 (SD)/surgery 80.56 ± 8.92 (SD)	7
Shin	Korea	2016	Retrospective	Full text	ESD 175/surgery 100	ESD (61.7 ± 8.7)/surgery (60.5 ± 9.7)	ESD 73.7%/surgery 73%	ESD 1.7%/surgery 1.0%	ESD major axis 12.4 ± 6.9 and minor axis 8.8 ± 4.9/surgery major axis 15.2 ± 8.7 and minor axis 12.0 ± 6.6	ESD 56 (range 45–58)/surgery 53 (range 44–60)	7
Soh	Korea	2016	Retrospective	Full text	ESD 55/surgery 27	ESD (52 (42–57))/surgery (53 (46–63))	ESD 38.2%/surgery 55.6%	N/A	ESD 15 (10–20)/surgery 27 (20–35)	ESD 30 (range 15–49)/surgery 20 (range 9–37)	7
Song	China	2015	Retrospective	Full text	ESD 29/surgery 59	ESD (65.3 ± 7.5)/surgery (45.8 ± 6.7)	N/A	ESD 7.0%/surgery 0.3%	ESD 27 ± 19/surgery 35 ± 16	ESD 26.9 ± 8.5 (SD)/surgery 22.3 ± 9.4 (SD)	7
Kim	Korea	2015	Retrospective	Full text	ESD 82/surgery 112	N/A	N/A	N/A	N/A	24	7
Fukunaga	Japan	2016	Retrospective	Full text	ESD 74/surgery 74	ESD (67.3 ± 8.8)/surgery (67.1 ± 7.5)	ESD 23%/surgery 21.6%	ESD 21.6%/surgery 18.9%	ESD 23.1 ± 10.1/surgery 24.7 ± 11.4	ESD 43.5 (IQR 26.3–76.0)/surgery 62.9 (IQR 36.5–91.7)	7

ESD: endoscopic submucosal dissection; SM1: tumor infiltration into the submucosal layer/500 *μ*m from the muscularis mucosae; SD: standard deviations; IQR: interquartile range; N/A: not available.

**Table 2:** The survival outcome of different studies.

	Ryu [19]	Shin [20]	Chiu [11]	Cho [12]	Fukunaga [22]	Kim [15]	Najmeh [17]	Park [18]
3 YSR (%) (ESD/surgery)	N/A	N/A	94.6%/89.7%	N/A	N/A	EC: N/A GC: N/A	N/A	80.0%/96.3%
OS (%) (ESD/surgery)	N/A	N/A	N/A	89.8%/90.0%	N/A	EC: 100.5 ± 1.3/84.9 ± 2.6 GC: 93.4 ± 3.2/85.8 ± 5.5 (month)	100%/90.3% (4 years)	N/A
5-year OS (%) (ESD/surgery)	100%/100%	N/A	N/A	N/A	97.1%/85.8%	EC: N/A GC: N/A	N/A	97.4%/96.1%
DFS (%) (ESD/surgery)	N/A	N/A	N/A	N/A	N/A	EC:93.6 ± 2.5/87.6 ± 2.0 GC: 89.7 ± 3.6/90.4 ± 3.5 (month)	84.6%/82.6%	N/A
5-year DFS (%) (ESD/surgery)	85%/97%	N/A	N/A	N/A	N/A	EC: N/A GC: N/A	N/A	N/A
5 YSR (%) (ESD/surgery)	N/A	92%/93.3%	N/A	N/A	N/A	EC: N/A GC: N/A	N/A	N/A
*P* value	5-year DFS: 0.001	0.496	0.44	0.565	0.01	EC:—OS: 0.397, DFS: 0.597 GC: N/A	N/A	3-year SR: <0.001, 5-year OSR: 0.280

ESD: endoscopic submucosal dissection; YSR: year survival rate; SR: survival rate; OS: overall survival; OSR: overall survival rate; DFS: disease-free survival; EC: expanded criteria; GC: guideline criteria. N/A: not available.

## Abbreviations

ESD: Endoscopic submucosal dissection
EGC: Early gastric cancer
SD: Standardized deviations
OR: Odds ratios
CI: Confidence intervals
QOL: Quality of life
LNM: Lymph node metastasis
DFS: Disease free survival
NOS: Newcastle–Ottawa Scale
Hb: Hemoglobin
EMR: Endoscopic mucosal resection
EGD: Esophagogastroduodenoscopy
CT: Computed tomography
LM: Lateral margins
VM: Vertical margins.
